# The Impact of Standing Electric Scooters on Maxillofacial Fractures: An Italian Multi-Centric Epidemiological Study

**DOI:** 10.3390/jcm13175195

**Published:** 2024-09-02

**Authors:** Giovanni Salzano, Francesco Maffia, Luigi Angelo Vaira, Roberta Fusco, Massimo Albanese, Salvatore Crimi, Marco Cucurullo, Fabio Maglitto, Claudia Maugeri, Marzia Petrocelli, Francesca Pitino, Paolo Priore, Fabio Roccia, Alessandro Tel, Anna Maria Baietti, Alberto Bianchi, Federico Biglioli, Chiara Copelli, Giacomo De Riu, Pier Francesco Nocini, Guglielmo Ramieri, Massimo Robiony, Valentino Valentini, Luigi Califano

**Affiliations:** 1 Maxillofacial Surgery Unit, Department of Neurosciences, Reproductive and Odontostomatological Sciences, University of Naples “Federico II”, 80138 Naples, Italy; 2 PhD Program of the Faculty of Medicine, University of Lisbon, 1649-028 Lisbon, Portugal; 3 Maxillofacial Surgery Operative Unit, Department of Medicine, Surgery and Pharmacy, University of Sassari, 07100 Sassari, Italy; 4 Oncology Medical and Research Development Division, Igea SpA, 80013 Naples, Italy; 5 Department of Oral and Maxillofacial Surgery, University of Verona, 37129 Verona, Italy; 6 Department of General Surgery and Medical-Surgical Specialties, School of Dentistry, University of Catania, 95124 Catania, Italy; 7 Operative Unit of Maxillo-Facial Surgery, Head and Neck Department, San Paolo Hospital of Milan, 20122 Milan, Italy; 8 Maxillo-Facial Surgery Unit, University of Bari “Aldo Moro”, 70121 Bari, Italy; 9 Department of Surgical Sciences, Division of Maxillofacial Surgery, Città della Salute e della Scienza Hospital, University of Turin, 10126 Turin, Italy; 10 Oral and Maxillo-Facial Unit, AUSL Bologna Bellaria-Maggiore Hospital, 40133 Bologna, Italy; 11 Department of Oral and Maxillofacial Sciences, Sapienza University of Rome, 00185 Rome, Italy; 12 Head-Neck and NeuroScience Department, Clinic of Maxillofacial Surgery, University Hospital of Udine, 33100 Udine, Italy

**Keywords:** electric scooters, craniofacial trauma, maxillofacial injury, traffic safety

## Abstract

**Objectives**: This study aimed to determine the impact of standing electric scooters on maxillofacial on the Italian territory. **Methods**: The authors analyzed the epidemiology of the injuries to define electric mobility’s impact on maxillofacial surgery practice. For this retrospective cohort study, data were collected by unifying the standing e-scooter-related fractures database from 10 Italian maxillofacial surgery departments. The reference period considered was from January 2020 to December 2023. The main data considered included age, gender, type of access, time slot of admission, type of admission, alcohol level, helmet use, dynamics of the accident, and area of the fracture. **Results**: A total of 79 patients were enrolled. The average age of the participants was approximately 31 years. The blood alcohol level was found to be above the Italian norm in 15 cases (19%). Only one patient wore a helmet. The most affected facial third was the middle one with 36 cases (45.5%), followed by the lower one (31, 39.3%). The most recurrent patterns were fractures of the orbito-malar-zygomatic complex (15, 19%), followed by multifocal (bifocal, trifocal) fractures of the mandible (14, 17.5%). **Conclusions**: This study demonstrated how maxillofacial fractures related to the use of electric scooters are associated with complex patterns, associated with a high rate of post-surgical aftermaths.

## 1. Introduction

Urban mobility is changing rapidly with the advent of electric micro-mobility (EM). EM is characterized by small, lightweight vehicles, operating at speeds of 25 km/h or 45 km/h [1]. Examples include standing scooters, bicycles, and segways, serving as practical alternatives to short-distance car journeys and valuable extensions to public transportation [2,3]. The introduction of e-scooter rentals on a global scale took place in the United States in 2017, while in Italy, the micro-mobility sector received an indirect boost during the COVID-19 pandemic [4,5,6]. The pandemic prompted people to seek alternatives to public transport, leading the Italian government to approve a bonus of up to EUR 500 for the purchase of e-scooters and other micro-mobility vehicles in May 2020. This operation ensured an even greater increase in the phenomenon, particularly in large cities such as Milan, Turin, Rome, and Naples [4,5].

Considering the spread of these new vehicles into existing road infrastructure, the pattern of road accidents and related injuries has changed [3,6,7]. Despite the ease of access to e-scooters for public use and their potential as a convenient and cost-effective solution for quick urban transportation, many concerns have been raised regarding the public health implications of e-sharing vehicle programs [6]. Specifically, there is a lack of attention on the incidence of traumatic injuries resulting from the unregulated and unsafe use of these services, particularly given the diverse urban environments where e-sharing apps operate [8,9]. Frequently, victims are individuals operating the scooters experiencing falls due to inadequate infrastructure or impact with other vehicles [10].

As the use of two-wheeled electric vehicles continues to rise, there is a growing warning about the associated increase in related accidents. Injuries predominantly occur in the head, upper limbs, and lower limbs, with less frequent involvement of the chest and abdomen regions [11]. A study found a significant increase of 222% in e-scooter accidents and injuries between 2014 and 2018 in the USA, especially in the last year of the period. Injuries ranged from minor cuts and bruises to severe trauma requiring intensive care. The number of hospitalizations due to these accidents increased by 365% in recent years [2]. Recent studies indicate that 26% to 58% of patients presenting with e-scooter-related trauma experience craniofacial soft tissue or bony injuries, posing additional challenges for the healthcare system [1,2]. Considering dental and maxillofacial hospitalizations, e-bike and e-scooter crashes accounted for more than 10% of admissions in Israel between 2014 and 2019 [12].

Accident-related traumas involving the craniofacial area pose particular concern due to the potential for damage to nearby vital structures, which can lead to fatal consequences [11]. Furthermore, impairment of sensory or motor functions in this region can significantly affect overall functionality. Moreover, the visible effects of facial injuries can result in profound psychological repercussions [13,14,15].

In these injuries, the adoption of helmets among e-scooter users is seldom higher than 5%, and additionally, many of the helmet varieties utilized offer limited protection for the facial bones [4,16]. Among the vehicles on the road, standing electric scooters (also known as standing e-scooters) represent a completely new vehicle, both in terms of driver position and conformation, while bicycles and electric scooters do not differ from normal motor vehicles. Risk factors and legislative implications for e-scooters may be comparable to those for bicycles, while the former is potentially more unsafe due to deceleration profile and stability [14]. E-scooter accidents have been shown to result more often in facial fractures, dental injuries, and facial soft tissue injuries than bicycle accidents [11].

Similar studies published in the literature have shown a prevalence of nasal, zygomatic, and mandibular fractures, related to facial trauma due to injuries implicating standing electric scooters [14,15]. More than half of the patients who reported this type of fracture were under the influence of alcohol, an important risk factor in this new phenomenon [17].

This study aimed to determine the pattern of maxillofacial fractures resulting from accidents involving standing electric scooters on the Italian territory, connecting different centers along the entire peninsula. The aim was to determine if fracture patterns reported in the literature are replicated within our country’s population. The authors also analyzed the epidemiology of the injuries, evaluated the circumstances of the accident, and completed medical records from admission to surgical treatment, to define electric mobility’s impact on maxillofacial fracture incidence.

## 2. Materials and Methods

### 2.1. Data Collection and Extraction

For this retrospective cohort study, data were collected by unifying an e-scooter-related fracture database from 10 Italian maxillofacial surgery departments, involved by personal invitation. Participating centers were as follows: “Aldo Moro” University of Bari, “Alma Mater” University of Bologna, University of Catania, University of Milan, “Federico II” University of Naples, “Sapienza” University of Rome, University of Sassari, University of Turin, University of Udine, and University of Verona. Following a geographical order, the centers were divided into North (Milan, Turin, Udine, and Verona), Center (Bologna and Rome), South (Bari and Naples), and Islands (Catania and Sassari). The reference period considered was from January 2020 to December 2023. Each participating center received a Microsoft Office Excel (Microsoft Company, Redmond, WA, USA, version 16.88, 2024) file as a detailed template for data collection to facilitate compilation. Before accepting and recording the individual questionnaires, the authors FMaf and GS clarified any discrepancies in the data by requesting further information and details in order to unify the data collected for each individual center. Data considered included the following: age, gender, type of access (ambulance AMB or own vehicle OV), time slot of admission (morning 8 am–2 pm, afternoon 2 pm–8 pm, or night 8 pm–8 am), day of the week, the season of the year, type of admission (day hospital or ordinary hospitalization), need for treatment in the emergency room, alcohol level (Italian reference limit >0.5 g/L), helmet use, dynamics of the accident (fall or collision with other object/car), the facial third where the fracture is located (upper “I”, middle/midfacial “II”, or lower “III”), other associated fractures, neurological involvement, soft tissue damage, the specific type of fracture, association with dental fracture, type of surgical treatment, and length of hospitalization. All files received were compiled into a single file for epidemiological evaluations of the individual parameters in the specific geographic area and the whole population.

### 2.2. Statistical Analysis

Quantitative variables were compared using the Mann–Whitney test (due to non-compliance with normal distribution in the Shapiro–Wilk test) and qualitative variables using Pearson’s Chi-squared test with Yates correction or Fisher’s exact test. Continuous data were presented as medians and percentile ranges. Multidimensional analysis using Multiple Linear Regression was used to assess the relationship between the most frequent fractures and individual factors such as sex, age, and alcohol consumption. The level of significance was set at = 0.05 for all analyses.

Statistical analysis was carried out with MATLAB R2023 (MathWorks, Natick, MA, USA).

## 3. Results

**Figure 1:**
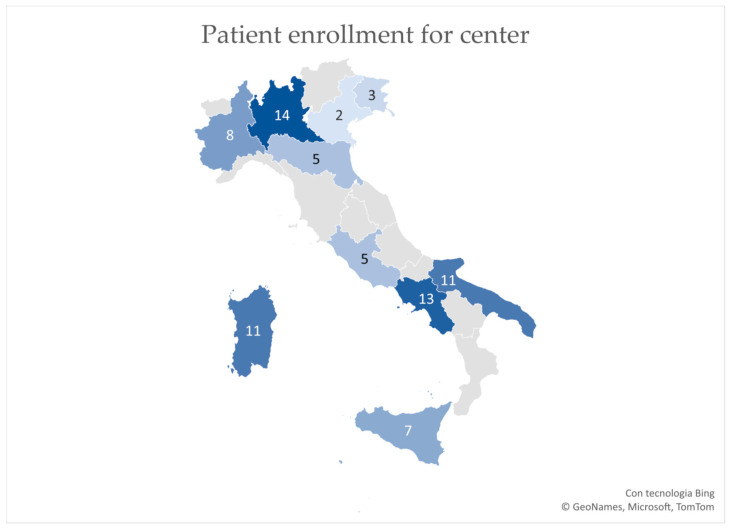
Colorimetric map of participating Italian regions. Color intensity changes according to the number of patients enrolled. The participant center home region has been colored entirely for graphical purposes.

A total of 79 patients were enrolled. The colorimetric map in Figure 1 shows the number of patients per single center. The geographical area with the most patients was the North with 27 patients (34%), followed by the South with 25 patients (31.5%) and the Islands (18, 22.5%), and lastly, the Center with 10 patients (12%). However, the distribution of fracture for the geographical area was not statistically significant (*p*-value > 0.05 for the Chi-square test).

**Figure 2:**
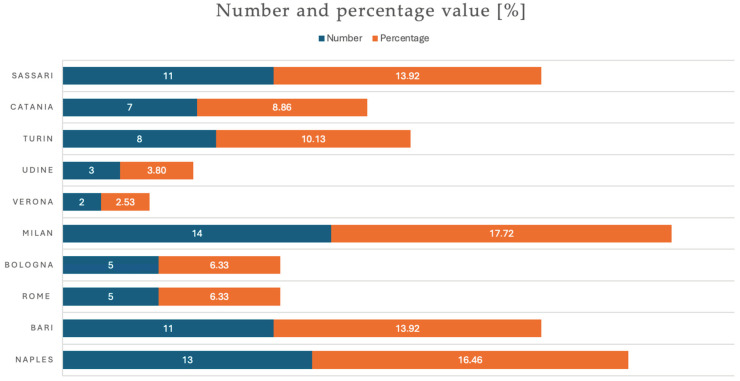
Distribution of patients by single city in absolute number and percentage.

Figure 2 reports the distribution of patients for a single city; there was a statistically significant rate among cities with prevalence in Milan (*p*-value for the Chi-square test = 0.003).

The average age of the participants was 31 years old. The M:F ratio was in favor of males, approximately 4:1 (82% M, 18% F). Two-thirds of the patients had access to the reference center by ambulance (53, 67%), while the remainder went to the hospital by direct access (26, 33%). The prevalence was statistically significant with a *p*-value for the Chi-square test equal to 0.0005. The distribution of the time slot showed a prevalence of nighttime hospitalizations (31, 39%), followed by the morning (25, 32%), and the afternoon (23, 29%).

There were no differences between weekdays and weekends, with an equal distribution (40 WD, 39 WE). Excluding spring (16, 20%), no major differences were recorded for the season of the year, with an equal distribution between winter (22, 28%), summer (21, 26.5%), and autumn (20, 25.5%). Most hospitalizations were on an ordinary basis (70, 88.6%), while only nine hospitalizations (11.4%) took place on a day hospital basis. About a third of patients required emergency room treatment (25, 31.6%), mainly represented by neurosurgical, ophthalmological consultations, and skin wound closures (*p*-value = 0.0007 for the Chi-square test).

The blood alcohol level was found to be above the Italian norm in 15 cases (19%). Only one patient wore a helmet. The dynamics of the accident mostly involved falls (61, 77%), while injuries related to impacts with objects or other vehicles amounted to 18 cases (23%).

**Figure 3:**
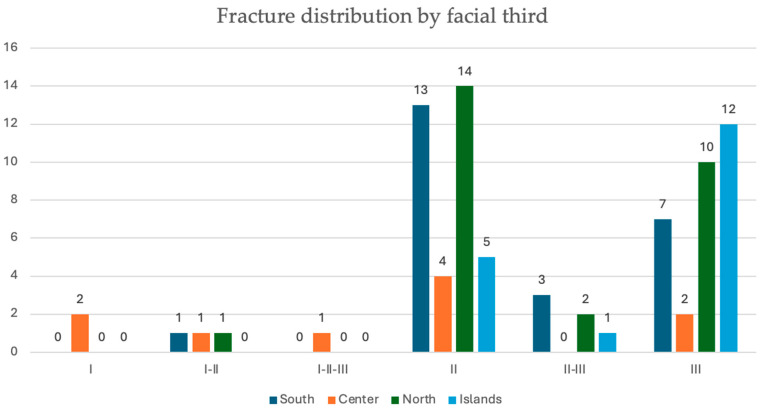
Distribution of fracture area (I, II, and III facial third) and their possible combinations by geographical macro-area (North, Central, South, and Islands).

The most affected facial third was the middle one with 36 cases (45.5%), followed by the lower one (31, 39.3%). The remaining cases were represented by combinations between the various thirds, the most represented of which was II-III (6, 7.6%). The most recurrent patterns were fractures of the orbito-malar-zygomatic complex (15, 19%), followed by multifocal (bifocal, trifocal) fractures of the mandible (14, 17.5%). Figure 3 reports the distribution of fracture area for the geographical macro-area. No statistically significant difference was observed in the Chi-square test.

In total, 11 patients (13.7%) reported other fractures during the trauma all of orthopedic origin with involvement of the upper limb. A total of 12 patients (15%) reported changes in neurological status: 10 losses of consciousness, and 2 subarachnoid hematomas. Nearly half of the patients sustained soft tissue injuries (37, 46.8%: *p*-value = 0.046 for the Chi-square test).

About a third of patients reported associated dental trauma or fractures (24, 30%). The average hospital stay was 4.62 days, with a range from 2 to 10 days. The fracture area characteristics for each geographical area are shown in Table 1.

Multiple Linear Regression showed that both age and alcohol consumption were statistically correlated with fracture area for geographical macro-area (see Table 2) with a *p*-value = 0.001 and a *p*-value = 0.02 for age and alcohol consumption.

## 4. Discussion

This study aimed to define the pattern of craniofacial fractures resulting from accidents involving standing electric scooters on the Italian territory, determining their impact on maxillofacial surgery practice. The simultaneous increase in their usage, coupled with the multitude of risk factors linked to riding e-vehicles has resulted in a rise in craniomaxillofacial injuries, imposing a significant burden on the healthcare system, particularly in emergency departments [6,14,18]. The rise of this phenomenon is global nowadays. The rate of hospitalizations in Israel resulting from incidents involving electric bikes and scooters surged by six times between the years 2013 and 2015 [14]. In the USA, between 2008 and 2017, 990 craniofacial injuries related to motorized scooters were documented, leading to an estimated 32,001 emergency department (ED) visits. The yearly incidence was observed to triple during this decade [15].

Numerous risk factors are related to the use of standing electric scooters. These factors include rider-associated factors such as demographic characteristics such as age and riding experience, environmental conditions like irregular surfaces and absence of dedicated lanes, vehicle-specific attributes such as reduced visibility (due to their compact size and quiet conduction), medium-elevated speeds, and inherent structural weaknesses due to lightweight construction, small wheel size, and limited or absent suspension systems compared to larger two-wheel counterparts like motorcycles [11,16]. Other important risk factors are represented by alcohol consumption and lack of helmet use. Regarding the lack of helmet use, the data in the literature and confirmed by our study are dramatic: in many studies, the percentage of use was less than one-third, with studies reporting the almost total absence of the protective device (Hoffeld et al. 1.7%, Toofany et al. 4.5%, Harbrecht et al. 0%, Benhamed et al. 6.1%, and Piccolino et al. 0%) [9,13,15,19,20,21]. The reasons may be many: lack of ride planning and improvised e-scooter rental, lack of helmet distribution by the provider despite being recommended before renting, and underestimation of danger [11,15]. Several studies analyzing electric micro-mobility reported an increased Abbreviated Injury Scale (AIS). Upper limb and head injuries tended to be more severe (abbreviated injury score ≥ 2) than other types of injuries (*p* < 0.05). There was an association between alcohol consumption and both helmet non-use (*p* < 0.05) and severe head and neck injuries (*p* < 0.001) [3,4].

The most popular electric scooter-sharing services in Italy are Lime, Bird, Bolt and Helbiz. On each of their sites are specific safety guidelines. Helbiz gives away helmets via an online form, but only in the USA or at presential events. Lime allows rentals with a 50% reduced speed “training mode” to ensure adequate experience before a full ride. Bolt offers insurance included in the rental for injuries due to the use of its scooters. Bird provides PDF guidelines on all rules of conduct. All providers recommend the use of the case according to current regulations in the country, although they do not make it mandatory or provide it with the rental. It is also advised to follow dedicated bike lanes where present [22,23,24,25]. As of today, 10 April 2024, the Italian government has determined to approve a new regulation to increase road safety: the use of helmets for electric scooters will be changed from recommended to mandatory, thus reducing the incidence of head-to-face trauma. [26] The new regulation is reported textually: “there will be new rules for electric scooters. Riders will be required to wear helmets and scooters must have license plates and insurance. Shared scooters will be restricted to designated areas. Strict penalties will be applied for illegal parking, riding against the flow of traffic, and using scooters on busy and dangerous roads outside of urban areas” [26]. This maneuver recalls the same action taken in 2000 when helmets became mandatory not only for motorcycles but also for scooters, with significant positive repercussions on traffic-related traumatology [21].

Alcohol use is a significant risk factor for craniomaxillofacial injuries due to standing e-scooter accidents, with Shiffler et al. reporting ten times increased risk of injury (5% to 53%) [17]. Examining studies in the literature, alcohol-related injuries resulting in head trauma were significantly greater than those of non-alcohol-related injuries [27,28,29]. Driving under the influence of alcohol increases not only rates of head injuries, but also internal organ injuries, and soft tissue lacerations. This evidence was noticed by Namiri et al. in their study on National Electronic Injury Surveillance System (NEISS) data: alcohol-related injuries were significantly associated with more severe outcomes, including head trauma, internal organ damage, and lacerations (*p* < 0.001) [27]. In their seven-year multi-centric retrospective review on e-scooter and maxillofacial fractures, Goh et al. highlighted how 86% of cases included were under the influence of alcohol at the moment of the injury [11]. In our study, blood alcohol levels were found to be above the Italian cutoff in one out of five patients (20%). Alcohol consumption was statistically correlated with geographical macro-area for fracture area with a *p*-value = 0.001. The reason why alcohol is such an important risk factor for accidents lies in its negative influences on visual and auditory functions, reaction time, attention, and vigilance [28].

The dynamics by which accidents occur on standing e-scooters are closely related to human reflexes and alertness since they are vehicles already constructed as unstable. Perception of instability or the sensation of falling activates neuromuscular protective reflexes, prompting the extension of the arms to moderate the fall’s impact and minimize injury severity. This reflexive action aims to safeguard various body regions, including the head, thorax, abdomen, and pelvis, by dispersing the force of impact [14]. The high rates of head and neck injuries associated with upper limb injuries suggest that drivers tried to break their fall before landing [4]. Alcohol intoxication leads to a suppression of the neuromuscular protective reflexes responsible for extending the limbs to cushion falls during accidents. This diminished reflex action increases the likelihood of direct trauma to the head, heightening the risk of facial bone fractures [10,14].

Some studies on maxillofacial traumatology due to electric micro-mobility vehicles have compared bicycle-related injuries with those due to e-scooters [1,8,20,27,30]. Benhamed et al. noted that standing scooter riders reported twice more frequently a severe head injury compared to bicyclists (24.2% vs. 19.9%). Furthermore, helmets were five times less used than bicycle riders (6.1% vs. 30.7%). Demographically, e-scooter patients were younger (24 vs. 29 years old), assuming that a smaller number of them possessed a driver’s license (50%), resulting in less familiarity with the rules of the road [20]. According to Italian national law, e-scooter drivers must be a minimum of 14 years old. However, Scquizzato et al., in their case series analysis of news reports about standing e-scooter crashes occurring in Italy from 1 January 2019 to 30 September 2020, reported that 7.6% (6/79) of e-scooter injuries involved individuals younger than this age cutoff [5]. In our population, only one patient (8 years old) was below the cutoff. Stray et al., in their cohort study, confirmed the pattern described by Benhamed et al. also adding that scooter drivers were also more often under the influence of alcohol (39.5% vs. 7.7%) and reported accidents more at night (87.2% vs. 47.2%) [20,30]. The data collected from our retrospective study match the trend described in the literature: most of the recorded traumas occurred at night (39%). This observation was statistically significant for the Chi-square test (<0.05). The risk factors related to this specific time of day are numerous and highlight an additional danger of these vehicles: apart from increased alcohol and drug intake during evening and nighttime, reduced visibility, fatigue, and decreased focus could also play a role in more accidents during these hours. Moreover, the quiet nature of most e-scooters may pose challenges for other road users to detect them in the dark [1,31].

Men comprised a higher proportion of individuals injured while riding both e-scooters and bicycles, aligning with previous findings. This disparity is likely due to gender variations in everyday tendencies toward risk-taking behavior [15,29,30]. This trend has also been observed in our population, where the M:F ratio was strongly in favor of men (4:1).

The majority of the studies analyzing the standing e-scooter phenomenon were conducted in Central Europe, where climate conditions vary across seasons, showing that e-scooter usage tends to be favored during warm and dry weather, increasing the likelihood of accidents during these times [1]. No pure prevalence among the four seasons was observed in our population. The explanation for this phenomenon could be the predominantly warm and milder Italian climate than the Central European climate. In addition, the inclusion of southern Italian centers facing the sea or located on islands, where the mild climate can extend over more seasons certainly influenced this result. Instead, the absence of a weekday-related pattern may be due to the presence of home-to-work-related accidents during weekdays, and leisure- and tourism-related accidents on weekends, with a preserved incidence of trauma associated with the phenomenon.

Considering the unstable structure of the standing electric scooter and road- and driver-related risk factors, the most common mechanisms of injury were falls, as also reported in several studies [8]. Benhamed et al. reported that 74.2% of e-scooter craniofacial injuries were related to falls, and only 25,8% were associated with collisions [20]. Scquizzato et al. described a different trend: most injuries were due to a collision with another vehicle (59%, 57/94) or a pedestrian (4%, 4/94). In half of the cases, the vehicle involved was a car [5]. In our retrospective analysis, falls were preponderant, with a ratio of almost 4:1 when compared with impacts against other vehicles or objects. Supported by the trends and risk factors already described, falls undoubtedly correlate with drunk driving, nighttime vehicle use, and underestimation of vehicle danger.

The etiology of craniomaxillofacial trauma is various, including road traffic accidents, beatings, and falls being the principal causes cited in the maxillofacial literature. The specific nature and site of the trauma are influenced by the circumstances. Typically, mandibular, then zygomatic, and nasal fractures are noted as the predominant types of facial injuries [14,15]. In all studies reporting analyses of maxillofacial fractures and electric scooters, the patterns were roughly always the same: orbito-maxillo-zygomatic (OMZ) complex fractures, midfacial non-OMZ fractures (zygomatic only, orbit only), nasal bone fractures, and mandibular multifocal fractures. These patterns are described with small variations in prevalence among them. Arbel et al. in their study analyzed the type of injuries comparing e-bicycles and e-scooters: while mandibular fractures were prevalent in both groups, e-scooter riders were more likely to suffer from condylar and subcondylar fractures (12.2% vs. 7.3%). Zygomatic complex fractures and NOE fractures were more frequent in the e-bicycle group [14]. Farajii et al. in their retrospective mono-centric analysis described a different trend: data revealed that the anterior maxillary sinus wall, nasal bone, and zygomatic bone were the most frequently fractured facial bones. In contrast, the mandibular subcondyle, mandibular condyle, posterior maxillary wall, and mastoid temporal bone were the least frequently affected [2].

The trend described in our study findings was different: midfacial fractures were the most common type of injury (45.5%, 36 cases), followed by lower third fracture (39.3%, 31 cases). Our population registered 15 cases of OMZ fractures (19%). A fact worth mentioning among the orbital fractures registered regards the only recorded pediatric patient (8 years old): the patient suffered a trapdoor fracture of the orbital floor. The mandible was the most involved bone, reporting different sub-patterns as bifocal, trifocal, and quadrifocal fractures. The most common mandibular fracture pattern was the combination of a parasymphyseal fracture with the contralateral subcondylar one (13 cases, 16.5%). Other combinations included angle and contralateral subcondyle and parasymphyseal and bilateral subcondylar.

The fracture patterns identified by this study and those in the literature are the two types that are most likely to expose themselves to postoperative sequelae: facial asymmetry, visual deficits, visible skin scars, malocclusion, tooth loss, and inflammation from osteosynthesis materials [14,15,16]. Piccolino et al. showed that nowadays, e-scooter injuries are the third cause of mandibular fractures after physical assaults and falls [21]. This may be due to the biomechanics of falling from a standing position with the inertia of being propelled forward. The high frequency of head and neck injuries alongside upper limb injuries suggests that drivers tried to cushion their fall before impact. Conversely, Bracher et al. and Hoffeld et al. found mainly craniofacial injuries, likely because many patients were alcohol intoxicated (91%) and thus unable to properly brace their fall with their hands [1,9]. Our study correlates partially with this association since most cases were craniofacial fractures only, while 11 patients reported associated fractures, which were all of the orthopedic type and involved the upper limbs such as clavicular, humeral, radial, ulnar, and scapular fractures. The association of soft tissue injuries and dentoalveolar fractures is a consistent trend in the literature [8,19,31,32]. Our casuistic recorded the incidence of this type of fracture in about 30% of cases (24/79), a percentage that is not very high but of great impact when considering that the teeth involved were the upper or lower incisors, with serious functional and aesthetic deficits. Almost half of our population reported at least one facial wound during the trauma, showing statistical significance. The dynamics of the fall, associated with moderate velocity, lead to significant impacts with the ground and with dragging of the face and an increased possibility of lacerations, abrasions, or contusions [2].

A possible limitation of the study is the different care pathways in the management of trauma patients in the various centers surveyed, with differences related to inpatient stay and type of access and admission. Although most accesses occurred via ambulance and with statistical significance, this may not reflect the real severity of the trauma. Another limitation is the lack of specific vehicle information (provider, actual speed, ground characteristics, and road rules of the precise location). The sample size is average, and despite this, studies on a larger population would allow for a deeper study of the characteristics of the phenomenon.

## 5. Conclusions

E-scooter craniofacial injuries are on the rise globally, and our analysis of recent years indicates this trend is present in Italy as well. This study highlighted how electric scooters are associated with patterns of moderate and severe maxillofacial trauma like mandibular and orbito-maxillo-zygomatic complex fractures, despite being medium–low-speed vehicles. Many of these cases are associated with weaknesses in the safety regulation of these vehicles, inadequate infrastructure, and incorrect behavior on the part of drivers, with underestimation of the danger and risk. We encourage the implementation of information campaigns alongside the widespread adoption of new micro-mobility vehicles. These campaigns would aim to educate users about safe driving practices and the potential risks associated with e-scooter riding, to reduce accidents and hospital admissions.

## Figures and Tables

**Table 1:** Characteristics for each geographical macro-area. BAC: blood alcohol content. Statistically significant values are reported in bold.

	South *n* = 24	Center *n* = 10	North *n* = 27	Islands *n* = 18	*p*-Value
**Age**	29.7	38.9	30.63	25.24	0.234
Gender (male, female)	20 M, 4 F	7 M, 3 F	23 M, 4 F	15 M, 3 F	0.93
**Type of access**					
Ambulance	24	4	12	13	**0.0005**
Own vehicles		6	15	5	
**Hospitalization time slot**					
Morning 8 am–2 pm	8	3	8	6	0.09
Afternoon 2 pm–8 pm	13	1	5	4	
Night 8 pm–8 am	3	6	14	8	
**Day of the week**					
Midweek	14	7	13	6	0.448
Weekend	10	3	14	12	
**Season of the year**					
Spring	6	1	6	3	0.99
Summer	7	2	6	6	
Autumn	5	3	7	5	
Winter	6	4	8	4	
**Type of hospitalization**					
Day hospital	19	9	25	17	0.51
Ordinary	5	1	2	1	
Need for treatment in the emergency room	7	1	16	0	0.0007
BAC	3	2	7	3	0.83
Use of helmet	0	1	0	0	
**Type of accident**					
Fall	19	8	21	13	0.99
Impact with another object/vehicle	5	2	6	5	
**Fracture area**					
I	0	2	0	0	0.547
I-II	1	1	1	0	
I-II-III	0	1	0	0	
II	13	4	14	5	
II-III	3	0	2	1	
III	7	2	10	12	
**Other associated fractures**	2	4	2	3	0.217
**Neurological involvement**	3	2	7	0	0.282
**Soft tissues’ injuries**	11	7	16	3	**0.046**
**Dental traumas**	6	5	8	5	0.765
**Duration of hospitalization**	4.13	4.2	4.25	4.47	0.873

**Table 2:** Multiple Linear Regression analysis.

Multiple Linear Regression	Coefficients	*p*-Value
Intercept	3.377	<<0.001
Age	−0.022	0.001
Gender	−0.212	0.285
Alcohol intake	−0.456	0.0178

## Data Availability

The data presented in this study are available on request from the corresponding author.
